# Engaging a Whole Child, School, and Community Lens in Positive Education to Advance Equity in Schools

**DOI:** 10.3389/fpsyg.2021.758788

**Published:** 2021-12-02

**Authors:** Sandra M. Chafouleas, Emily A. Iovino

**Affiliations:** ^1^Collaboratory on School and Child Health, Institute for Collaboration on Health, Intervention, and Policy, University of Connecticut, Mansfield, CT, United States; ^2^Department of Educational Psychology, Neag School of Education, University of Connecticut, Storrs, CT, United States

**Keywords:** whole child, equity, multi-tiered frameworks, prevention science, developmental systems approach, ecological systems framework, school development program

## Abstract

Recent decades of education policy, research, and practice have brought focus on a positive education approach as applied within tiered service delivery frameworks to meet diverse needs of varied intensities. Related, the science of implementation has begun to increase understanding of supports to strengthen use of a positive education approach within tiered service delivery frameworks. To date, the body of work has fostered important shifts in how problems are viewed and addressed using a positive lens, supporting more equitable opportunity in education. To realize the full potential, however, there is a need to integrate theory and science as embedded within a whole child, school, and community lens. We propose that positive education will advance equity when grounded in integrated theory and science across developmental systems theory, prevention science, ecological systems theory, and implementation science. We first provide a brief overview of schools as a context to serve as assets or risks to equity, followed by a discussion of theory and science using a whole child, whole school, and whole community lens. We end with directions for science and practice in advancing a positive education approach.

## Introduction

Positive education encompasses a broad range of theories and orientations, with common elements including proactive problem-solving, promotion of student well-being, and positive relationships (Halliday et al., 2020). Several examples of positive education initiatives that engage these elements exist in United States schools. Social and emotional learning (SEL) and positive psychology interventions, for example, are strengths-based options with their focus on building resilience, building relationships, and promoting self-regulation (Halliday et al., 2020). In addition, positive behavior interventions and supports (PBIS) may be conceptualized as a positive education approach. PBIS focuses on promoting positive child outcomes through proactive strategies (e.g., behavior-specific praise and positive practice of schoolwide expectations) that are universally available to all children. Another example might be found in positive youth development programs given attention to building positive developmental settings and promotion of well-being (Clonan et al., 2004).

Expanding on the common elements of positive education as offered in these examples, we also view positive education as requiring incorporation of systems thinking to enable a shared purpose toward well-being promotion. Kern et al. (2020), for example, proposed systems informed positive psychology as a way to expand the reach of positive psychology given the interconnectedness of individuals, others, and the environment and the dynamics between those elements. Perspectives of individuals within the system, who is invited in and who is excluded, how the system adapts over time, and how systems organize to come together are attended to in systems thinking (Kern et al., 2020). It is this sort of interconnectedness, which we propose describing as a whole child, school, and community lens, that is needed to enable positive education to advance equity in schools.

Recent decades have brought policy, research, and practice agendas with focus on a positive education approach as applied within tiered frameworks for organizing and delivering tiered services. Grounded in prevention science, the foundation to tiered frameworks is provision of evidence-based core services to all, with data to drive proactive identification of gaps in expected performance and decisions regarding supplemental supports. Related, the science of implementation has begun to increase our understanding of factors that strengthen effective use of these tiered frameworks. To date, the body of work has fostered important shifts in how problems are viewed and addressed, but the comprehensive integration of a positive education approach within these frameworks has yet to reach full potential in advancing equity in schools.

Actualizing the full potential of positive education to advance equity requires integration of theory and science as embedded within a whole child, school, and community lens. This lens is theory-driven at its foundation, with explicit connection to integration across bodies of literature. In this article, we propose that a positive education approach will advance equity when grounded in integrated theory and science across (a) developmental systems theory, (b) prevention science, (c) ecological systems theory, and (d) implementation science. We start by setting up the need for integrated theory and science with a brief overview of schools as a context to serve as assets or risks to equity. Next, we organize discussion of theory and science within a whole child, whole school, and whole community lens. We begin with the whole child, using developmental systems theory as the predominant focus. We then move to the whole school, with a focus on prevention science and applications in education through multi-tiered systems of support. Finally, we discuss the whole community, using integration of ecological systems theory and implementation science as keys to effective and sustained effort. We then offer directions for science and practice to fully enable a positive education approach to advance equity in schools.

## Schools as Assets or Risks to Equity

School is a critical setting for public health intervention, with child development and learning central to closing gaps in education outcomes and reversing negative health outcomes throughout life (Comer et al., 2004; Hahn and Truman, 2015). Newly released data from the United States Centers for Disease Control and Prevention (CDC)’s Youth Risk Behavior Survey, for example, have noted connections between academic achievement and health behaviors such as sleep, alcohol and tobacco use, physical activity, and nutrition (Centers for Disease Control and Prevention, 2019). In their critique, Hahn and Truman (2015) argue that education is a fundamental social determinant of health, offering grievous illustrations. The authors illustrate that in the United States, for example, a man with a graduate degree could be expected to live 15 years longer than one with a high school education. A dose response appears between years of education and many health-related behaviors – for example, for those with less than 9 years of formal education, there is a higher prevalence of risk behaviors (Hahn and Truman, 2015).

The positioning of school as a public health intervention stems from our understanding of the contributions within and connections across the many contexts that shape a child’s development. Within Bronfenbrenner’s (1979) ecological framework, school is placed within the system most directly connected to the child, the microsystem, appearing right along with family, peers, neighborhood, and religious institutions. As Osher et al. (2020) noted, school settings have potential to “enhance developmental range, buffer the effects of stress and trauma, promote resilience, and accelerate the development and integration of affective, cognitive, social, and emotional processes (p. 9).” These features in school contexts serve as critical assets to healthy human development, and when available, enable potential for equity in education. When unavailable, the setting features are risks – that is, incongruent with positive, healthy human development given insufficient support and potential mismatch with development or culture. In addition, chronic exposure to these incongruent features in a school context can lead to habituation, thus perpetuating a cascade of negative educational outcomes. In this way, the school context serves as a risk to equity.

**FIGURE 1:**
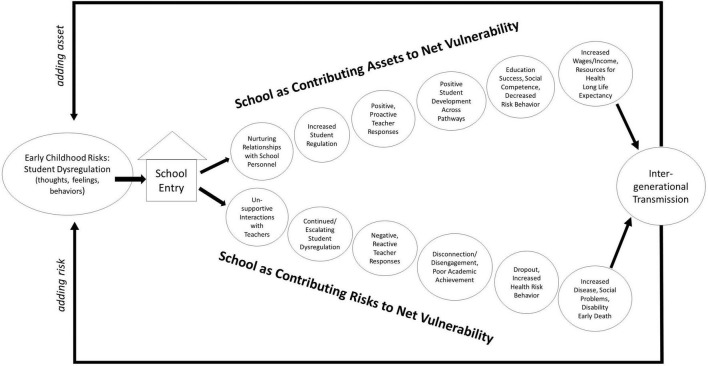
Illustration of developmental cascades: School as an asset or risk to net vulnerability.

When connecting contexts that shape child development, Osher et al. (2020) discuss the goal of minimizing net vulnerability. Positive human development occurs through enabling contexts in ways that minimize risks and maximize assets. As previously noted, schools are an important context to a child’s net vulnerability; however, schools contribute to the asset and risk balance of vulnerability in uneven ways across children. In this way, education environments serve as contributors to inequity. Discussion of school as an asset or risk should not be separated from interactions with other contexts, both present and historical. The cumulative consequences of these interactions and transactions within and across systems have been referred to as developmental cascades (e.g., Masten and Cicchetti, 2010). An illustration with focus on school as an intervention for facilitating negative or promoting positive cascades can be found in Figure 1. In this example, one path presents school as posing risk, adding to the child’s net vulnerability and thus perpetuating inequity that extends intergenerationally. A related example can be found in education policy reform related to school discipline, which was enacted to improve education outcomes yet has resulted in pervasive exclusionary disciplinary practices that not only are disproportionate for certain demographic groups (e.g., students who are Black, boys, with disabilities) but also disconnected from emerging science about adverse childhood experiences (e.g., Chafouleas et al., 2021).

Attempts to enable the school environment as a contributor to assets (i.e., reduced net vulnerability) exist, yet there is potential missed emphasis on the theory underpinning the what and the why. Although it may be tempting to ask why theory matters if the initiative works, doing so can set up challenges to interpretation of outcomes and perhaps, most importantly, sustainability (Pianta and Walsh, 1998). As one example, we return to discussion of exclusionary discipline as a serious problem, thus prompting a quest for promising alternatives. In a comprehensive review of the literature on alternative options to school discipline, Welsh and Little (2018) summarize the overall body as promising, albeit nascent, yet also potentially problematic given disconnect from theory. Mismatch between theory of action behind alternative options and causes of disparities may be present, particularly regarding opportunity to engage an ecological lens. For example, their review yielded that most alternative options focused on “within” child intervention, such as strategies to assimilate into the school culture. Overall, the various alternatives reviewed tended to focus intervention in a particular area, such as teaching skills to students, restoring relationships after problem behavior, addressing biases and teaching cultural-responsiveness, or restructuring the system. These alternatives each sound promising but may not maximize sustained positive outcomes or implementation.

In their recommended directions for future work, Welsh and Little (2018) suggest that disparities in exclusionary discipline are undertheorized, and they recommend working toward an integrated theoretical framework that expands upon student problem behavior to also include issues of race and culture in intervention as well as discrimination and bias. We certainly agree, adding that such an integrated framework must also be connected to the science of development and learning to maximize potential for equity in school environments. Integration across theories and science is key to advancing schools as contributing assets to each child’s net vulnerability, which is the foundation for enabling equitable positive education environments.

Next, we expand on our perspective to building and refining an integrated framework to advance equity in positive education as grounded in a whole child, school, and community lens. Integrated theory and science is needed, or in other words, unpacking the “whole” is needed.

## Whole Child: It’s Not Just Academic

We begin with the whole child as the individual sits at the center of the ecosystem, meaning that the intended outcomes of interventions delivered in any system are ultimately intended to support healthy child development. The big question that drives school-focused intervention, perhaps, is regarding what defines successful child development. The past two decades of education policy reform in the United States defined educational outcomes as driven by academic indicators, with specific focus on increasing achievement for students who are of color, living in poverty, English-language learners, and with disabilities. For example, although policies such as the No Child Left Behind Act of 2001 (Congress.gov, 2001) drew initial praise for calling attention to “achievement” gaps, the resulting narrow focus on reading and math test scores coupled with punitive school accountability did not yield expected results (e.g., Darling-Hammond, 2007; Darling-Hammond and Cook-Harvey, 2018).

Calls have been made to use the science of development and learning to overhaul school policy and practice toward a whole child lens in education. Although seemingly a recent phenomenon, the roots of a whole child lens were planted over 50 years ago in work seeded by Dr. James Comer. Comer’s school development program (SDP) establishes child development as the cornerstone to learning, with six interacting developmental pathways through which brain maturation occurs (Comer et al., 2004). Comer’s developmental pathway framework includes physical, cognitive, psychological, social, ethical, and language. See Table 1 for a definition and key features of each of Comer’s developmental pathways, with corresponding alignment of converging descriptions in more recent initiatives such as the ASCD Whole Child Approach (ASCD, n.d.); Learning Policy Institute Whole Child Education (Learning Policy Institute, n.d.); Aspen Institute Commission on Social, Emotional, and Academic Development, 2018; and the University of Connecticut Collaboratory on School and Child Health.

**TABLE 1:** Description of Comer’s six developmental pathways, including alignment with other whole child approaches.

UConn collaboratory on school and child health	Learning policy initiative’s whole child education	Aspen Institute Commission on Social, Emotional, and Academic Development (2018)	Comer’s school development program*	
			
			Pathway	Key features
Physical	Physical health		Physical	Goal: Acquire knowledge about physical development and use it to make decisions that lead to healthy development. Examples include physical health, nutrition, and responsible decision making
Academic	Cognitive development academic development	Academic	Cognitive	Goal: Increase capacity to analyze, synthesize, and evaluate information; achieve mastery in content areas; problem-solve effectively; and enjoy learning. Examples include flexible thinking, skill at manipulating information and the environment
			Language	Goal: Increase capacity for receptive and expressive language, used appropriately across contexts. Examples include interpretation of non-verbal cues, understand spoken and written communication, and effectively use spoken and written communication
Emotional	Mental health social emotional development identity development	Emotional	Psychological	Goal: Develop capacity for self-regulation, management of emotions, and positive sense of self. Examples include self-awareness, self-worth, competence, and emotion regulation
Social		Social	Social	Goal: Build and maintain healthy relationships, across diverse characteristics and settings. Examples include interact well with others and effective communication in relationships
Behavioral			Ethical	Goal: Acquire knowledge of and demonstrate appropriate behaviors, be just and fair, and make decisions that promote well-being of self and others. Examples include respect for rights of others, and integrity of self

*Adapted from Comer (2020) and Comer et al. (2004). The ASCD whole child approach is not mapped here as the description provided is a whole child approach (as opposed to a whole child), which includes ensuring the child is safe, healthy, supported, engaged, and challenged.

The premise behind a whole child lens is embedded in developmental pathways – in other words, this means enabling every child to reach their full potential by providing appropriate and supportive interactions with adults who help them along their paths and across domains of development. Comer advocated that school provides a universally accessible setting in which enough adults are available to promote development along all pathways (Comer et al., 2004), thus positioning the mission of education as whole child. The roots to a whole child lens lie within a developmental systems approach. Fueled by trans-disciplinary work (e.g., developmental psychology and molecular biology), a developmental systems approach emerged in the second half of the 20th century. As noted by Molenaar et al. (2013), the overarching assertion is that “developmental processes are explained as the result of self-organizing processes with emergent properties that have complex, dynamic interactions with environmental influences (p. 3).” In other words, a developmental systems approach calls attention to the shared contributions of genes, environment, and epigenetic factors – with research focus on identifying the mechanisms in development.

Moore (2016) elaborates through a comparison of the developmental systems approach and the analysis of behavior. The author shares the limitations of a nature versus nurture perspective given the importance of temporal dynamics, or interactions across factors that form a single complex system, in human development. Importantly, that system is defined not only by the current presentation but through the contributions of historical factors. Past events can contribute to current behaviors, and as such, it is important to recognize historical antecedent factors. Behaviors themselves, however, are heavily influenced by proximate factors and interactions, calling for critical focus on the relationship in order to enable proximate causes of behavior that stem from provision of positive supports that are developmentally and culturally matched.

In their use of a developmental systems lens to present cautions in applying the emerging research on resilience in schools, Pianta and Walsh (1998) borrowed from the metaphor of the branching tree as presented by Sroufe and Rutter (1984). To understand the genesis and routes of adaptation in development, pathways to outcomes can be traced by following ways in which branching occurs. Branches are not supposed to grow evenly across time and can grow in different directions. However, growth is foundationally dependent on nurturing, with provision of early intervention when branches are not taking the desired path.

In human development, nurturing is the developmental relationship. In fact, relationship has been defined as the foremost active ingredient in establishing the positive, supportive contexts across interventions. Li and Julian (2012) operationalized the developmental relationship as “enduring emotional attachment, progressively more complex patterns of joint activity, and a balance of power that gradually shifts from the developed person in favor of the developing person” (p. 157). The authors further argue that the developmental relationship should serve as the foundational metric for evaluating the quality and potential impact of intervention – that is, every program, practice, and policy decision should be evaluated based on facilitators or barriers to developmental relationships.

Returning to the application of developmental pathways within the school context, Pianta and Walsh (1998) shared that:

In our view, a singular focus on success stories, whether they be at the school or individual level is the tendency to overlook the fact that success develops and any effort to promote success by replicating its correlates is likely to fail. It is no accident that Comer’s model for school reform (e.g., Comer, 1996), which has withstood a reasonable amount of scrutiny and replication, is called the “School *Development* Program” (emphasis added) (p. 409).

The authors further note that intervention attempts must start with the understanding that outcomes take time to develop and involve a process of multiple influences that may be potentially uncontrollable. As we discuss later within whole community, desirable conditions for positive and healthy development include strong relationships that enable supportive bonds and interactions among influences.

When disconnected from developmental relationships, trauma-informed schools and SEL are examples of promising education practices that could face challenges to both sustained implementation and outcomes. The past decade has brought a surge in interest to bring the principles of trauma-informed care into school settings (Chafouleas et al., 2021), with the goal of ensuring that all staff are informed about trauma and understand the importance of their role in providing a safe and nurturing environment that avoids re-traumatization. The promise of trauma-informed schools is tightly connected to developmental relationships yet has not always been explicitly front and center in efforts to date. Recent analyses have suggested that the bulk of existing literature has focused on (a) building staff knowledge about trauma and its impact with less attention to roles in responding to trauma or fostering school environments that avoid perpetuating traumatization, and (b) trauma-specific intervention delivered to students with the goal to reduce trauma symptomology (Chafouleas et al., 2016, 2021; Gherardi et al., 2020). As such, the full promise of a whole child lens in positive education has yet to be fully realized.

The second example, SEL, has brought tremendous effort to restructure what students are taught, moving away from heavy emphasis on academics to include the skills or habits needed to successfully navigate throughout life (e.g., self-awareness, perspective taking, capacity to recognize and regulate emotions, and relationship skills). Again, the promise of SEL is connected to developmental pathways, but it must be made explicit as to how the skills fit within the whole picture of child development. In addition, programs to teach defined social-emotional skills may be well-intentioned, but outcomes may not be realized in the absence of integration of context, culture, and relationships.

Strong potential exists to enhance integration with trauma-informed principles and advance equity through merging of current work in SEL into transformative SEL. Although just beginning to emerge in the literature, transformative SEL connects SEL with salient social identities (e.g., race and culture) and self-concept (Chafouleas et al., 2021). These directions align with implications for schools as identified in a recent meta-analysis on motivation across different academic achievement, well-being, goal orientation, and persistence-related student outcomes (Howard et al., 2021). In brief, the authors recommend school emphasis on autonomy-supported teaching practices, which are congruent with developmental relationships and a positive education approach.

As illustrated in both examples, this new wave of education reform in the United States holds tremendous promise to integrate what often appears as parallel initiatives; however, these efforts must be explicitly connected back to the why of the work, which is founded in a whole child lens. In the absence of a whole child focus, such as when attention is directed toward academic goals or building skills without contextual match, inequity will likely continue as an outcome.

In summary, a developmental systems approach provides a foundation for understanding the what and the why behind a whole child focus in positive education. Efforts such as the Comer SDP have been successful given the placement of relationships and the associated science of development and learning at the center of the work. Picking up on Comer’s position that school is a universally accessible setting to promote development along pathways, strong positive relationships serve among the most critical resources offered in the school environment. Thus, we now turn to review of the whole school, meaning how do we ensure that the structures are in place to establish adult knowledge, skills, and attitudes necessary for supportive connection that assists each child at the most appropriate branches in their developmental pathways. As we argue, the knowledge, skills, and attitudes cannot be considered separately from the systems in which they are expected to be used. Thus, our whole school focus is on the frameworks for organizing the work of positive education delivery in schools, and supporting adults in actionable work to select, deliver, and evaluate education services that are developmentally and culturally appropriate.

## Whole School: the Promise of Tiered Systems

Research over the past several decades have shown that risks and, ultimately, lasting negative outcomes, can be prevented. Progress in this line of research can largely be attributed to the growth of the interdisciplinary field of prevention science. Emerging in the late 1990’s through fields such as epidemiology, education, human development, and psychopathology, “[the] overarching framework of prevention science is a public health model for the conduct, design, and sequence of research and intervention strategies,” (p. 1, Stormont et al., 2010). Burns (2011) summarized the principles for prevention science originally presented by Coie et al. (1993) to include: addressing the developmental processes that can lead to negative outcomes; addressing predictors of negative outcomes through intervention before they stabilize; prioritizing intervention for individuals at high risk for negative outcomes; and coordinating action across systems and domains of functioning. Typically, a prevention-based model is described as offering universal strategies for all individuals within a particular population regardless of individual risk; targeted strategies which target individuals who may be at risk; and select strategies for high-risk individuals who have detectable signs of challenges. In sum, the premise of prevention science is to systematically mobilize resources, engage measures sensitive to identifying concerns early (e.g., any gap in expected performance), and put in place appropriate services to mitigate those risks (i.e., reduce or eliminate gaps in expected performance).

In K12 education settings in the United States, contemporary application of tiered service delivery is more commonly referred to as multi-tiered systems of support (MTSS). MTSS frameworks have generally been described as having three tiers of service delivery intensity: those focused on all students (Tier 1: preventing challenges before they occur); some students (Tier 2: mitigating challenges for those at risk for or exhibiting early challenges), or a few students (Tier 3: intensive supports to reduce challenges for those who did not respond to Tier 1 or 2 supports). MTSS frameworks offer schools a structure for organizing processes to provide a continuum of supports for students based on identification of a gap between expected performance and actual performance (Lane et al., 2016).

A cornerstone of effective MTSS is not only the availability of a continuum of supports from prevention to intervention, but assessments that focus on identification of who needs which supports and monitoring of responsiveness to the provision of those supports. Thus, a fully functioning MTSS framework includes ongoing links between assessment and intervention. Decisions regarding services are made through data-informed processes to match evidence-based practices to identified needs, whether it be intensive services for an individual, targeted services for a specific population or need, or services for the entire student body. At the systems-level, implementation data are collected to determine whether initiatives are being delivered as planned. Universal indicators (e.g., screening assessments, attendance, referrals, and grades) are used to identify gaps in performance and provide population surveillance over time, with data examined at regular intervals.

**FIGURE 2:**
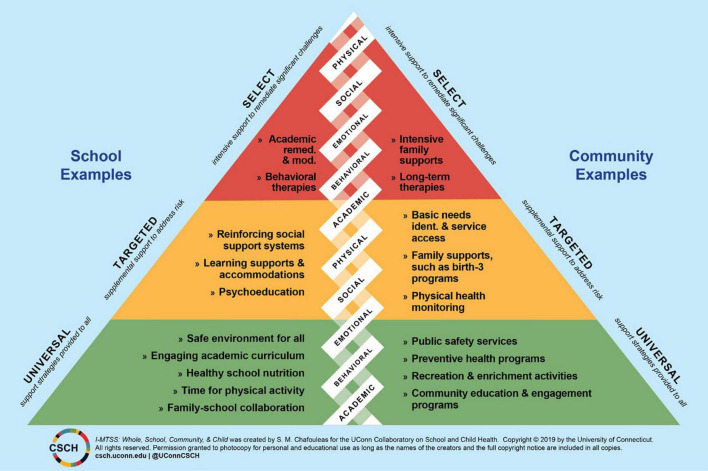
Visual example of multi-tiered systems of support that integrates whole school, community, and child (Chafouleas, 2019). Image reproduced with permission from the Collaboratory on School and Child Health at the University of Connecticut.

To date, MTSS in schools has historically involved a siloed approach to supporting student needs. In fact, initial applications of MTSS in schools were focused on academic skills, which then extended to support behavior (Walker et al., 1996; Lane et al., 2016). In recognition of the missing support across other areas of student development (e.g., social) and the challenges that arise from siloed academic and behavior tiered systems of support, shifts to comprehensive and integrated tiered systems of support have initiated. Models such as the comprehensive, integrated, three-tiered model of prevention (Ci3T) include tiered academic, behavioral, and social-emotional systems to support students more effectively and efficiently (Lane et al., 2016). As an example, see Figure 2 for a visual of MTSS that integrates domains of functioning and shares example services across tiers and settings.

Recently, integrated MTSS (I-MTSS) has been conceptualized as a component of promoting social justice, as it can facilitate equitable access to education for students who are often marginalized, such as students with emotional and behavioral disorders (Melloy and Murry, 2019; Chafouleas et al., in press). When done well, an integrated tactic to implementing tiered systems of support is aligned with promoting equity in schools (Melloy and Murry, 2019). Chafouleas et al. (in press) discuss that a key component of MTSS done well is gathering data from a variety of sources (e.g., internal student factors, home variables, and classroom management practices) to aid in effective decision-making around student supports. However, the authors also point out gaps in educator skills related to use of data in decision-making, along with gaps in broader support for data-based decision-making (Chafouleas et al., in press).

As such, we pose the question: are MTSS truly making education more equitable for all students, regardless of ability, race, ethnicity, socioeconomic status, culture, language, etc.? Avant (2016) positioned that MTSS implementation must change to align with a socially just, equitable lens, with proposed changes such as preparing educators’ social competence, ensuring curricula and practices address diversity, and establishing a culture of social justice starting with school leaders. We agree, adding that establishing equitable school environments also requires connection with factors beyond the school microsystem. The previously mentioned SDP connects these factors and engages a positive and preventive approach in fostering equitable education environments.

The SDP was first introduced in 1968 by Dr. James Comer. SDP, which is a process for comprehensive school improvement, was developed using principles from public health, social relationship theory, and child development (Comer et al., 2004). SDP theory is congruent with positive education, and SDP practices are aligned with school-based prevention models given emphasis on whole school change, enabling schools to serve as a hub for supporting child development, family involvement in school-based teams and decisions, and ongoing professional learning for teachers and school staff (Comer et al., 2004). The SDP process includes guiding principles, operations, and teams designed to foster a school climate where teachers can teach, and students can learn. Similar to MTSS, the SDP process was not designed to be one more thing for schools to do, but rather to assist schools with better managing, organizing, integrating, and aligning curriculum, instruction, and assessment, and engaging in data-driven decision making to prevent negative outcomes for students through supporting child development, particularly for minoritized youth (Comer et al., 2004). SDP provides a framework for stakeholders to communicate effectively and plan for school improvement through prioritization of prevention efforts.

Parallels exist between MTSS and SDP, including a focus on prevention and meeting diverse student needs through evidence-based strategies and data-driven decision making. However, explicit emphases in the SDP process are tied to the previously described whole child lens. As first promoted in SDP over 50 years ago, evidence for “non-academic” influences such as child self-concept, self-efficacy, teacher-student relationships, and parent involvement with potential to accelerate student achievement continues to grow (Hattie, 2017). Although some MTSS frameworks address these malleable child and environmental factors, they are not typically explicitly woven into MTSS procedures. It is this gap that offers strong potential for positive education to contribute to reducing inequities.

In sum, whole school contributes to the rationale and organizing mechanisms for determining what is needed and for whom. It is important to ask, however, whether current MTSS practices are in fact equitable in the absence of grounding in a whole child lens. To move forward in sustainable effort, integration of the larger ecological context, or the whole community, with whole child and whole school is needed.

## Whole Community: Drivers to Sustainability

Whole community acknowledges the need to connect whole child goals across settings and contexts to maximize outcomes and bolster sustainability of efforts. As noted by Kern et al. (2020) in their proposal to apply systems thinking to positive education, intervention targeting one part of a system without consideration of interrelatedness across the entire system has potential to result in unintended consequences. As we outline, positive relationships, including among adults connected to the child, continue to undergird the ultimate success of any initiative, both within and across child-serving systems (family, school, and community). In addition, success is contingent on moving through stages of implementation – again, ensuring that all stakeholder voices are informed and engaged through phases. These conditions are important to enable the current implementation context, but alone may not be sufficient for sustainability. Thus, in this section, we define whole community as merging ecological systems framework and implementation science to engage cross-ecosystem strategies that can heighten enduring use of a positive education approach.

Throughout the article, we have made nods to the relevance of cross-microsystem collaboration in enabling positive developmental pathways. Comer (2020) emphasizes that whole child work must be contextually driven, that is, co-constructed with all stakeholders and building from strengths in the current context. Drawing from the natural context and employing purposeful programming for generalization can yield better student and implementation outcomes than attempts to import manualized programs into the current environment (Riley-Tillman and Chafouleas, 2003; Clonan et al., 2004).

The Comer SDP principles are based in supportive relationships, which extend beyond child-adult to adult-adult interactions. As aptly stated, “In every interaction you are either building community or breaking community,” (p. 148, Comer et al., 1996). Guiding principles include no-fault (everyone is accountable, focus is on problem-solving over blame), consensus (decision-making to build consensus about what to try), and collaboration (responsiveness by all, from leaders to team members; Comer et al., 2004). Full implementation of SDP is described as a process that occurs over multiple years across phases (pre-orientation, orientation, transition, operationalization, and implementation). In short, SDP brought forth important elements that can be viewed today in contemporary terms used to describe key features to adoption, uptake, and successful implementation of intervention, such as “buy-in,” “phases of implementation,” and “usability.”

School development program has a long history of implementation in schools and districts across the United States. Multiple studies over the past decades have supported significant student outcomes across domains of achievement, behavior, and attitudes. In fact, the Comer SDP process was identified as one of few effective models in a meta-analysis of school reform efforts (Borman et al., 2002). SDP represents an example for which the intervention ingredients for the “secret sauce” are in place for successful outcomes and implementation within the school microsystem. The SDP theory of change positions that SDP facilitates these outcomes by buffering child risk contributed by external factors through direct effects on classroom factors and indirect factors associated with school organization, climate, and culture.

Although the language may not fully align, the work was visionary in setting the stage for the application of implementation science in education innovations. The challenges encountered by SDP and related reform efforts are not about producing expected child outcomes but centered on long-term sustainability of efforts. Factors such as phases of implementation and user-centered design are in place to enable successful implementation within the current context of the microsystem (i.e., school) and interactions among microsystems (i.e., school and family), but there is more to be mapped in theories of change to bolster sustainability. Guidance for these maps to enduring change may be drawn from implementation science frameworks.

As noted by Lyon and Bruns (2019), multiple iterations of implementation frameworks have emerged that have application to school settings, with an important commonality being the need to attend to multiple layers to bolster success. These layers or factors that obstruct or enable change have been described as implementation determinants. Many individual determinants are possible, which can be grouped into categories such as outer setting, inner setting, bridging factors, and the intervention itself – along with acknowledgment of interconnections, interactions, and linkages across the categories (Moullin et al., 2019). The inner setting, for example, refers to the immediate context or school microsystem, and may include factors such as principal leadership; individual teacher interest in, knowledge about, and skill with intervention delivery; resources to support implementation such as time or coaching; and data systems for evaluating decisions. Complete descriptions of each group can be found in Moullin et al. (2019).

Returning to our understanding that implementation occurs in phases, McIntosh et al. (2014) noted that different factors may be more or less important to driving success at different stages of implementation. In their work to understand variables least and most important for initial implementation and sustainability to School-Wide Positive Behavior Supports, some factors such as school administrator support, implementation fidelity, and staff buy-in were indicated as facilitators across stages. In contrast, district-level support was perceived as critical to sustainability, and parent involvement was less critical during initial implementation yet was very important to sustainability.

To date, limited research attention has been directed toward understanding influences of the outer setting, specifically those factors that facilitate sustained implementation. Lyon and Bruns (2019) define the outer setting as “the larger political, social, and economic context in which implementation occurs. This includes norms, laws, and broader policies as well as interorganizational linkages within a larger service system,” (p. 208). In schools, this can mean the district, state, and federal levels. In their discussion of translation and use of evidence in mental health intervention, Atkins et al. (2016) advocate that in order to effectively respond to child vulnerability, dissemination and implementation research agendas must be intentional in aligning with an ecological model. That is, whole community contributions to advancing equity in the school microsystem hinges on building capacity to define determinants across levels in the ecosystem that facilitate sustained implementation over time.

As previously noted, both intended and unintended consequences can result from initiatives put in place at each layer within the ecosystem (Kern et al., 2020). In the example provided by Lyon and Bruns (2019), for example, unfunded or underfunded mandates require shifting and are often put in place without *a priori* consideration as to what resources will be re-allocated away from something else in order to comply with the mandate. In another example, Clonan et al. (2004) remind us that adding classroom activity (e.g., 15 min SEL curriculum) means taking something else away (e.g., reduced time for recess). Given that advance understanding of that impact often is unknown, there is rationale to both modify the natural context to the least extent possible in intervention design as well as establish steps to evaluate intended and unintended outcomes. Together, both examples illustrate the need to align goals, expected outcomes, and values within and across each system to establish points for synergy in advocacy and commitment to enduring change.

This whole community alignment occurs through integration with whole child and school. The whole child lens gives us child development and learning theory in which to ground the work in a positive education approach. We have learned that sole focus on academics does not get to desired outcomes, yet a pendulum swing to SEL may not yield desired outcomes in the absence of grounding within a whole child lens. In the United States, work to do so is being put forth by groups such as the Center for the Developing Child^1^, which uses the science of child development and learning to enable positive developmental cascades (i.e., whole child lens) within a whole community lens as applied to early childhood. Similar directions to ground effort in a whole child lens can be expanded to the K12 public school. The whole school lens identifies school as a critical microsystem for facilitating public health goals in prevention and promotion of desired child and life course outcomes. Whole community is then used to tie together whole child and whole school to advance equitable outcomes for all. For example, see Figure 2.

As summarized by Osher et al. (2020), advancing equitable education outcomes requires support and efforts across the general public and political leaders, policy that promotes evidence-based whole child practices, quality implementation, formative assessment to monitor progress, scaled uptake, and explicit goals around equity and cultural competence. As such, there are “provocative opportunities for defining and studying an increasingly intentional constructive enterprise between children, the ecologies in which they grow and learn, and the relationships to the adults and peers in their lives and, by doing so, open pathways for new creative approaches to solving seemingly intractable learning and social problems,” (p. 24, Osher et al., 2020). Given its tenets, positive education has strong potential to contribute to advancing equitable education outcomes when a whole child, school, and community lens is used to advance a comprehensive and integrated positive education approach.

## Discussion: Moving Research and Practice Forward

In this article, we propose that a positive education approach must be embedded within a whole child, school, and community lens to advance equity in schools. Such an approach is theory-driven with explicit integration across bodies of science. Specifically, we define a whole child, school, and community approach as grounded in integration across (1) developmental systems approach, (2) prevention science, (3) ecological systems framework, and (4) implementation science. For over 50 years, Dr. James Comer has championed nurturing of the whole child through the foundational roles of developmental relationships and no-fault consensus collaboration among adults who care for them. Comer’s work is tied to the developmental systems approach, and connects to prevention science with emphasis on nurturing development as requiring proactive (versus reactive) service delivery.

In recent decades, prevention science in schools has evolved into multi-tiered systems of service delivery that address targeted domains of functioning (i.e., academic and behavior), perhaps losing focus that whole child success necessitates attention to nurturing of many developmental pathways in different ways across stages of development. The ecological systems framework brings emphasis to interactions within and across systems that influence outcomes across generations. The school system is not alone in contributing to net vulnerability but can serve as a critical asset to child development – yet it has not historically done so for every child. Each child brings forth a unique set of risks and assets, and thus, for schools to contribute to reducing net vulnerability necessitates strong positive relationships with adaptation of services to the individual need. Implementation science bolsters potential capacity for promising practices to contribute assets by maximizing features of usability and sustainability within intended settings. Together, a whole child, school, and community lens sets the stage to enable the full potential of a positive education approach to advance equity in schools.

In recent reflections on his personal experiences and history of the SDP, Comer (2020) notes that

…many schools, through no fault of their own, are not prepared to adequately promote [student] development and learning. The school challenge exists, in part, because knowledge regarding how to intentionally design, organize, and manage schools in ways that support student’s development, learning, and increased opportunity for life success is not adequately understood and embedded in the adult population (p. 43).

This next generation of positive education research and practice can advance the foundations built by Comer to enable a sustained whole child, school, and community lens that puts equity at the center of the work, disrupting the history of school as contributing to negative developmental cascades. Education training, practice, and research has long-embraced the multi-faceted and often complex array of factors contributing to child well-being, and now is the time to advance equity through elevation of the what and why in comprehensive and integrated services to enable sustained impact. Positive education should not be relegated to a surface level program geared to fix an immediate need; efforts must be steeped in integrated science and theory directed toward long-term sustained outcomes.

Much of the groundwork in positive education has been laid, and our next phase in science and practice can be used to fulfill the vision that every child has access to supports that are developmentally and culturally appropriate. When we frame equity as the overarching mission to a positive education approach, the activities of school are embedded in a whole child lens which emphasizes developmental relationships, and service delivery is driven by a prevention orientation and informed through the science of implementation to bolster sustainability.

## Author Contributions

All authors listed have made a substantial, direct, and intellectual contribution to the work, and approved it for publication.

## Conflict of Interest

The authors declare that the research was conducted in the absence of any commercial or financial relationships that could be construed as a potential conflict of interest.

## Publisher’s Note

All claims expressed in this article are solely those of the authors and do not necessarily represent those of their affiliated organizations, or those of the publisher, the editors and the reviewers. Any product that may be evaluated in this article, or claim that may be made by its manufacturer, is not guaranteed or endorsed by the publisher.

## Footnotes

1
https://developingchild.harvard.edu/
